# Knickpoints in Martian channels indicate past ocean levels 

**DOI:** 10.1038/s41598-019-51574-2

**Published:** 2019-10-22

**Authors:** Sergio Duran, Tom J. Coulthard, Edwin R. C. Baynes

**Affiliations:** 1 Department of Geography, Geology and Environment, University of Hull, Hull, UK; 2 Department of Civil and Environmental Engineering, University of Auckland, Auckland, New Zealand

**Keywords:** Geomorphology, Hydrology, Inner planets

## Abstract

On Mars, the presence of extensive networks of sinuous valleys and large channels provides evidence for a wetter and warmer environment where liquid water was more abundant than it is at present. We undertook an analysis of all major channel systems on Mars and detected sharp changes in elevation along the river long profiles associated with steep headwall theatre-like valleys and terraces left downstream by channel incision. These breaks in channel longitudinal slope, headwalls and terraces exhibit a striking resemblance with terrestrial fluvial features, commonly termed ‘knickpoints’. On Earth, such knickpoints can be formed by more resistant bedrock or where changes in channel base-level have initiated erosion that migrates upstream (such as tectonic uplift or sea level change). We observed common elevations of Martian knickpoints in eleven separate channel systems draining into the Martian Northern lowlands. Numerical modeling showed that the common elevations of some of these knickpoints were not random. As the knickpoints are spread across the planet, we suggest that these Martian knickpoints were formed in response to a common base level or ocean level rather than local lithology. Thus, they potentially represent a record of past ocean levels and channel activity on Mars.

## Introduction

**Figure 1:**
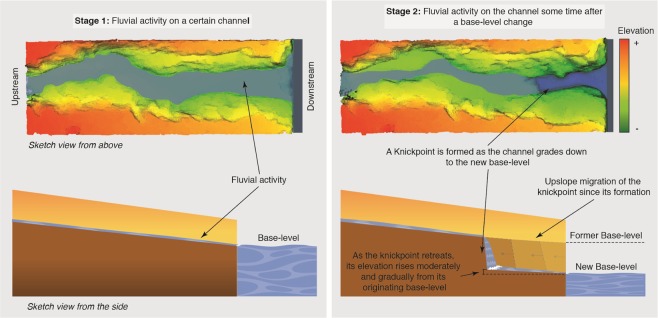
Conceptual diagram illustrating how base-level change knickpoints represent dynamic locations recording elevations of the base-level. The left part of the figure represents, seen from above and from the side, fluvial activity on a channel ponding into a base-level. The right part of the figure represents how, subsequent to a change in the base-level, the fluvial activity grades down the channel surface to the elevation of the new base-level. On the top-right figure, the knickpoint marks the boundary in between the part of the landscape adjusted to the new conditions (downstream the knickpoint) and those that are yet to adjust (upstream the knickpoint). On the bottom-right figure, it shows how the knickpoint is formed and migrates upslope as the channel grades down to the new elevation of the base-level. Modified from reference 8 and used under a Creative Commons Attribution 4.0 International License (https://creativecommons.org/licenses/by/4.0/). We produced the mosaic in this figure using Adobe’s Illustrator CS6 software (https://www.adobe.com/es/products/illustrator.html).

The surface of Mars contains many channels, meanders belts and deltas that may have been formed by flowing water^1–3^. Within these channels, there are dramatic drops in the channel longitudinal profile often associated with large theatre-shaped headwalls^4,5^. On Earth, such features are termed ‘knickpoints’^6^ and can be formed as the result of fluvial erosion over layers of different resistance bedrock^7^ or where an abrupt fall of the base-level (e.g., sea level) generates a vertical offset in the channel bed profile^8^. Terrestrial knickpoints are also characterized by their upstream migration over time that can leave abandoned terraces and downstream meander cutoffs^9–11^. The phenomena of a knickpoint propagating upstream and its dynamics have been scrutinized in many field, numerical and physical modelling studies^12,13^. Typically, terrestrial base-level change knickpoint erosion has been shown to be triggered by an uplift of the river bed^14^, a change in the river base-level^15^ or an uplift in coastal areas effectively leading to relative sea-level changes^16,17^. Common elevations of knickpoints in different streams can indicate a regional forming mechanism, e.g. the movement of a tectonic fault or a drop in relative sea-level^8,18^ as seen in Fig. 1, rather than lithological controls^18^. Furthermore, knickpoint formation and migration can be extremely rapid in response to very large flood events, as well as to unexceptional floods^19,20^.

Decades of satellite exploration on Mars revealed a planet extensively dissected by many fluvial-like channels. Beginning in the Southern highlands and extending down to the Northern lowlands, some channels exceed a thousand kilometers in length and drop several kilometers in elevation^21,22^. These channel systems have few tributaries but contain theatre-shaped head walls and major knickpoints – sometimes referred to as ‘cataracts’ or ‘inner channel headcuts’, – which previous research has suggested to be caused by episodes of bedrock erosion by high-discharge flows^22,23^. The geographic distribution, orientation and termination of these flood-eroded channels in the Northern lowlands suggest they once flowed into a standing body of water or ocean^24,25^. Subject to considerable discussion, the presence of an early Northern ocean is also supported by the estimation of the water needed to carve the Martian valley network^26^, its abrupt termination^27^, the identification of possible shorelines^25,28^ and the subsequent explanation of variations in shoreline topography^29,30^. The identification of tsunami deposits in the Chryse Planitia and north-western Arabia gives support to the presence of such a body of water at an elevation of ca. −3,795 m earlier in the Late Hesperian at 3.6 Ga^31^. In addition, deltaic deposits^32^ provide evidence for a large standing body of water in the Northern lowlands at an elevation of −2,540 ± 177 m around 3.4 Ga ago (around the latter stages of the Late Hesperian, ages herein based on Neukum chronology^33^). Whilst the current surface conditions on Mars do not support the presence of liquid water^21^, whether Mars hosted an early Northern ocean and the subsequent evolution of oceans/bodies of water are of significant importance for understanding the early Martian climate and the transition towards the current hydrologic and environmental conditions.

Here, we have analyzed the major channel systems spread across Mars using a systematic and automated procedure to identify channel knickpoints likely formed by base-level changes. These knickpoints are geographically widespread and fall into four common elevation zones with tests showing the lower two have a likelihood of being formed by random processes of less than 5% (see Supplementary Material). This suggests that the lower two knickpoint elevation zones were formed either under periods of two stable ocean levels or during two or more phases of fluvial activity coupled with changing Northern ocean levels.

## Methods

For this analysis, we selected only large Martian channels with widths greater than 1 km, with no obvious external disruptions to the channel network (e.g. significant craters) and those that flow from the Southern highlands into the Northern lowlands. We covered all longitudes and the location of the channels studied is shown in (Supp. Fig. 2). We used a semi-automated method to identify potential knickpoints by means of the Mars HRSC MOLA Bended global 200 m resolution DEM. Channel networks were highlighted using a flow accumulation method, then a series of MATLAB based tools (see Materials and Methods supplement) that identified breaks in longitudinal channel slope as potential knickpoints. The bottom (lowest) elevation of each knickpoint was then recorded, since it represents the elevation of the contemporaneous ocean level. Each knickpoint was visually checked for features frequently found on terrestrial base-level change knickpoints – e.g. those formed by the movement of a tectonic fault or a drop in relative sea level – and ranked according to how many of these identifiers were present. Such identifiers are: the presence of upslope channel, the identification of vertical incision downstream of the knickpoint, the identification of abandoned terraces downstream of the knickpoint and the identification of incised meanders downstream of the knickpoint (Supp. Figs 4–6). For visual inspection, we used HiRISE (High Resolution Imaging Science Experiment) images of the region where the knickpoint is located. In areas non-covered by HiRISE, we used the Context Camera (CTX) images. On Earth, as base-level change knickpoints migrate upslope, their elevation rise gradually from their base-level as seen in Fig. 1. Thus, knickpoints generated from the same base level change in separate valleys may have slightly different elevations, so to identify any commonality in the elevation of base-level change knickpoints, we need to look over a range of elevations. Therefore, we assigned a Gaussian Kernel Function for each knickpoint and, subsequently, summed these Kernel functions providing us with a continuous planet-wide Kernel Density Estimate of base-level change knickpoints per elevation (Supp. Fig. 3). This enabled us to identify at which elevations these knickpoints are clustered that we termed knickpoint zones (Supp. Fig. 1). To test the non-random significance of the Kernel Density values displayed in our record, we bootstrapped our sample of 34 knickpoints producing repeated (1,000) resamples with replacements. We assessed whether the clustering of our measured knickpoints were significantly non-random by comparing our record with the bootstrap distribution of resamples (Supp. Fig. 9) which showed the whole record was not significantly non-random. Subsequently, we split our data into knickpoints from zones 1-2 and for 3-4 and carried out the same analysis on each data set (Supp. Figs 10 and 11). These analyses showed that the subsamples for zones 3–4 were significantly non-random, exceeding by some margin the 95% confidence level (Supp. Figs 11 and 12).

## Discussion

**Figure 2:**
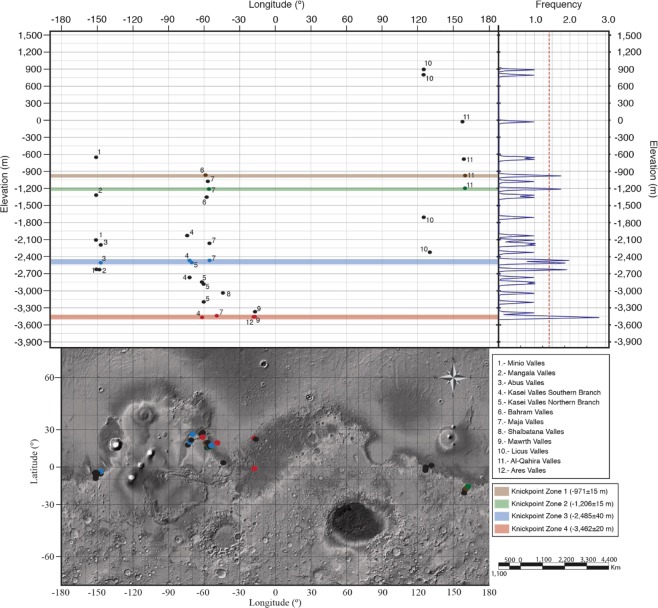
Topography, distribution of base-level change knickpoints and elevation of knickpoint zones on Mars. The lower map shows the topography of Mars with superimposed possible base-level change knickpoints categorized by the knickpoint zone in which are included. The upper part of the figure comprises, on the right, the Kernel Density Estimate of base-level change knickpoints by elevation and, on the left, a graph displaying the distribution by elevation of the possible base-level change knickpoints against their longitude. Both graphs share the same elevation axis (Y-axis). In the upper-left graph each knickpoint is associated with a number indicating the channel system within is located. The upper-left graph and lower map share the same longitude axis (X-axis). The knickpoints contained within each knickpoint zones have been represented by a point with the same colour than the knickpoint zone. Knickpoints represented by the black colour are not contained within any knickpoint zone. Some of the 34 potential base-level change knickpoints are located too close to each other and thus overlap at this map scale (see Supplementary Table 1 for the complete list of knickpoints).

**Figure 3:**
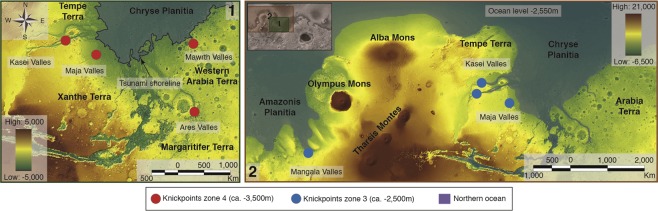
(**1**) View of the circum-Chryse highland-lowland boundary region, which consists of Chryse Planitia, Western Arabia Terra, Tempe Terra, Margaritifer Terra and Xanthe Terra. This boundary is breached by the circum-Chryse outflow channels, where Kasei Valles Maja Valles Ares Valles and Mawrth Valles exhibit base-level change knickpoint (red points) at −3,500 m. The shoreline mapped by tsunami deposits, and the body of water comprised, is represented in blue color. **(2)** View of the circum-Chryse and Sirenum Terra highland-lowland boundary region, where a Northern body of water at −2,550 m (defined by the average elevation of deltaic deposits) is represented by color. Kasei Valles, Maja Valles and Mangala region channels exhibit base-level change knickpoints (blue points) at −2,500 m. The images for both panels (1 & 2) are color-coded shaded-relief MOLA digital elevation models (460 m/pixel). Credit: MOLA Science Team, MSS, JPL, NASA. We produced the mosaic and maps in this figure using Esri’s ArcGIS 10.6 software (http://www.esri.com/software/arcgis).

34 potential base-level change knickpoints were identified in 12 major channels spanning the full width of the planet and covering elevations from 900 to −3,500 m, as shown in Figs 2 and 3. None of the channels had equilibrium long profiles, all base-level change knickpoints are located along the Southern edge of the Northern lowlands and every channel contains (at least) one potential base-level change knickpoint.

Our analysis shows four clear elevation zones where there are more than one base-level change knickpoint with the lower two zones having frequencies greater than 2 and 3 respectively, indicating they are unlikely to be random (Fig. 2). Importantly, the lowest two zones have knickpoints from geographically distant channels – thereby making it less likely that the common knickpoint elevation was due to a common geological control (e.g. resistant lithology at the same elevation). Therefore, we suggest that the knickpoints were formed as the non-equilibrium channels adjusted their long profiles to a common – planet wide – base level, as sketched in Fig. 1 and evidenced in Fig. 3. Whilst we cannot completely rule out that the knickpoint zones are due to other controls, we argue that the most likely explanation is that the common knickpoints capture past ocean/sea levels.

Importantly, in support of this explanation, the elevations of our knickpoint zones correspond with ocean levels identified in previous research. The detection of deposits likely emplaced by tsunami waves^31^ at an elevation of ca. −3,795 m suggesting an ancient shoreline (during early stages of Late Hesperian) in the same regions where knickpoints in zone 4 are located at the elevation of −3,462 ± 20 m. The identification of deltaic deposits^32^ found in widespread locations provides evidence for a large standing body of water (during the latter stages of the Late Hesperian) at an elevation of ca. −2,500 m, thus fully consistent with the past ocean/sea level inferred from the knickpoint zone 3 at −2,485 ± 40 m. Whilst this latter ocean level has been questioned by recent investigations around the Gale Crater^34^, the similarity in elevation of our knickpoint zone 3 and deltas within other regions closer to them^32^ (e.g. Tempe Terra or circum-Chryse Region) suggests that (at least) some of these deltaic deposits were most likely formed by global ocean controls. Importantly, our methodology of using knickpoints, for the first time, enables the independent identification of multiple base/ocean levels within the same record.

Further supporting our argument that knickpoints zones 3 and 4 represent past ocean levels, the timing of flows within channels where knickpoints for zones 3 and 4 occur correspond with the dates of corresponding elevation shoreline/tsunami deposits. For knickpoint zone 4, studies indicate the Kasei Valles, Ares Valles, Maja Valles and Mawrth Vallis formed or were active around 3.6 Ga ago^35–38^ concurrent (in geological time) with the emplacement of tsunami deposits^35^ and the Deuteronilus Shoreline^39^. For our knickpoint zone 3, studies show periods of flow in the Kasei Valles and Maja valles^35,40^, as well as channel formation in Mangala Region^23,35^ around 3.4 Ga ago.

Therefore, we suggest the following scenario led to the formation of these major channels and their knickpoints. (1) At around 3.6Ga an ocean or major water body was in place at c. −3,500 m (2) Major flows occurred in the Kasei, Ares, Maja and Mawrth Valles, carving both the channels and also incising into the non-equilibrium channel long profile generating knickpoints at or close to the base level (ocean level) (3) Flow stopped in these channels and over the next 0.2Ga ocean levels rose to c. −2,500 m (4) A second major period of channel flow occurred in the Kasei and Maja Valles, as well as in the Mangala channels, leading to channel incision and the development of new higher knickpoints close to the new base level.

Morphological evidence in the channels suggests that flows were (relatively) short lived. Firstly, the elevation of the bottom of knickpoints can show a small rise in elevation as they retreat upstream from the originating base level (e.g. Fig. 1). In our examples the relatively small difference in elevation between the knickpoint zones and the corresponding shorelines/ocean levels suggests that these knickpoints did not retreat long distances (compared with the scale of these channels), which indicates their forming events may have been short lived or ephemeral. Secondly, most terrestrial knickpoint research is based on the deviation of a channel long profile from an equilibrium profile^41^ and, visually, our long profiles are clearly not at equilibrium (Supp. Fig. 8), as confirmed using long profile/drainage area analysis (see Supp. Material). This may be explained by the Martian water being sourced differently from Earth channels^42^, but can also show that such channels flowed for a period insufficient to achieve any equilibrium, thereby supporting episodic or short periods of flow. It is important to note that an equilibrium channel is not a precondition for the presence of knickpoints generated by base level change, as evidenced by those observed by Mackey *et al*.^43^, Germanoski & Ritter^44^ and in the laboratory experiments of Baynes *et al*.^18^. Thirdly, the preservation of the lower knickpoints following base level rise suggests that the base/ocean level rose very rapidly, or more likely during a period of no channel flows. Therefore, our results suggest major channel and ocean activity during the Late Hesperian, with two distinct phases of significant channel flows at 3.6 and 3.4 Ga interacting with a Northern ocean at −3,500 m (3.6Ga) rising to −2,500 m (3.4Ga).

A fundamental requirement for separate channel base-level knickpoints to have preserved the signal of the same base level fall is for there to have been no uplift or subsidence of the surface after the knickpoint formation. However, the emplacement of the Tharsis complex has likely caused a major impact on the Martian topography^29,30^ as can be seen in the Supp. Fig. 7. Our knickpoint zones 1 & 2 are within areas of negligible/no surface elevation changes, allowing them to precede (or be concurrent with the early stages of) Tharsis emplacement and still preserve their initial elevation. In contrast, our knickpoint zones 3 & 4 are within the area affected by Tharsis, but their geomorphology (e.g. Kasei Valles incises back into regions constructed by Tharsis volcanism) suggests that they formed after Tharsis emplacement (or during the latter stages of). The topographic distribution of the knickpoints within the knickpoint zone 3, being consistent with deltaic deposits^32^, implies no significant modification of the Martian topography since their formation. Therefore, they suggest a post-Tharsis ocean level consistent with a large portion of the Arabia shoreline^28–30^, which (in turn) indicates a re-occupation – during the late Hesperian – of the older Arabia ocean level (presumed Noachian in age^29^). However, variations in such shoreline topography requires further studies on the relationship between the evolution of a Northern ocean and the Tharsis volcanism.

At its simplest level, knickpoints are markers of (any) disequilibrium within a channel profile, and can be caused by many reasons (e.g., lithological boundaries, base level fall, periodic flow, etc.). Here, we show (i) there are knickpoints on Mars, (ii) the identified knickpoints are at consistent elevations across the Northern part of the planet and not randomly grouped, (iii) they are located close to known past ocean levels, both in elevation and location, (iv) channels with corresponding knickpoints have flowed at the same time as shoreline records were deposited and (v) the knickpoints are not associated with lithological boundaries. This evidence leads to two options for interpretation: knickpoints could either have been formed by (1) a drop in ocean level and the channels grading to that new base level or (2) periodic switching on of the fluvial network and grading to whatever the ocean level is at that moment in time. Given the known chronology of the ocean elevations (i.e., a rise), the fact the channels are not in equilibrium, and the known occurrence of short periods of channel activity (e.g. outburst floods), this favours interpretation 2.

We quite deliberately avoid discussing mechanisms for channel flow or ocean formation and acknowledge that our findings may complement^45^ or contradict^46^ other research. However, our results are consistent with a warmer and wetter early Mars climate and suggest that, at stages in Mars’ history, massive channelized flows interacted with Northern oceans at two distinct levels (as proposed in previous investigations^47^). Furthermore, these findings indicate a complex, dynamic hydrosphere with an active hydrological cycle and an ocean exerting a global control on channel systems.

## Supplementary information

Supplementary information
