# Supplementary material for: Knickpoints in Martian channels indicate past ocean levels
Source: Sci Rep. 2019 Oct 22;9:15153. doi: 10.1038/s41598-019-51574-2 (PMC6805925; doi:10.1038/s41598-019-51574-2)
Supplement: Supplementary file 1 — Supplementary information [file 41598_2019_51574_MOESM1_ESM.docx]

SUPPLEMENTARY MATERIALS AND METHODS

Overview of methodology for identifying knickpoints in the Martian channels

This section describes the methodological approach adopted to identify knickpoints in Martian channels. This methodology follows four steps outlined below and in Suppl. Figure 1:

1. Selection of the channels

2. Detection of knickpoints

3. Identification of base level change knickpoints

4. Comparison of knickpoint elevation and distribution across the planet.

**1. Selection of the Martian Channels**

Channels were selected according to the following criteria: All longitudes were examined, covering only exogenic–like channels beginning in the Southern highlands and ponding into the Northern lowlands are eligible. We rejected those channels with major disruptions (as for example craters) within their longitudinal profile. Due to limitations in resolution of the DEM and remote sensed data, we also rejected channels with a width on average less than 1km. For channels with tributaries, we analysed only the main trunk stream. Table 1 displays the channels selected.

**2. Detection of knickpoints**

The knickpoint detection was carried out on the Mars HRSC MOLA Blended DEM Global 200m provided by the U.S. Geological Survey. The map projection is Simple Cylindrical and the total elevation uncertainty of the product is at least ± 3m. The Digital Elevation Model (DEM) was imported in Arc-GIS, where channel networks were extracted. For this, we generated a sinkless raster by using Arc-GIS filling functions, computed the direction of the flow and the accumulated flow. To define a proper drainage line, an empirically tested value of 1% of the maximum accumulated flow was used as a threshold to distinguish channels. The main trunk of the channel network was isolated, defining the topographic elevation -3,950m as the bottom end of the channel. In cases where the base of the channel was not at this elevation, we extracted a minimum channel length of 1,200km. To prepare the main trunk of the channel to be exported and to aid subsequent tests, we split the channel into 3km reaches. Sensitivity testing using different length reaches/sections indicated that the average slopes of the 3km reaches have sufficient accuracy to detect knickpoints whilst removing some noise in the DEM. We added a point at the end of each reach and, finally, we extracted the elevation all of these points from the original DEM and the sinkless DEM to generate two channel longitudinal profiles. We conducted the same procedure for each of the channels selected.

These data were then imported into excel. Firstly, each reach of the channel was numbered following the numerical sequence displayed in the equation 1. We assigned a value equal to 1 to the reach at the most upstream part of the channel. By using the information from the sinkless DEM, we estimated the longitudinal channel slope of each reach as the difference in elevation between its initial and final point divided by its length, except in the first reach of the channel in which the slope was automatically defined as 0. We assigned a slope equal to 0.0001 to those reaches with slope inferior or equal to 0.

We created a table with the number of the reach, its longitudinal slope and its distance upstream (km). By using the information from this table, we identified the elevation of the bottom surface of each knickpoint as a reach in which the average of its slope and the slope of the three previous reaches is greater than 0.003, being none of those previous reaches an already detected bottom surface of a knickpoint, and coupled with a slope smaller than 0.001 for the following two reaches. We visually inspected the results provided by the algorithm, assuring that it detected every significant break in longitudinal slope. Finally, we obtained the elevation of the reach identified as bottom elevation of the knickpoint from the original DEM.

This first stage generated a database with more than 150 possible knickpoints. The database comprises the ID number of the knickpoint, the name of the channel system in which was identified, the coordinates of the reach in which the bottom surface of the knickpoint is located, its elevation, upstream distance from the bottom surface of the knickpoint to the upstream most part of the channel, multiple high resolution aerial images (CTX and HiRISE) of the area in which each knickpoint is located and, finally, the elevation of the bottom surface of the knickpoint extracted from the original DEM. We obtained the coordinates of the reach in which the bottom surface of the knickpoint is located by exporting that reach from ArcGIS into Google Earth as KML and extracting its coordinates.

nj = ni + 1 (1)

Where nj is the number of the considered reach and ni is the number of the reach upstream

**3. Identification of base-level change knickpoints**

The assessment of whether these breaks in longitudinal channel slope could have been formed by variations in their base-level is of fundamental importance. Therefore, each knickpoint was assessed individually – and globally.

The individual assessment was performed on each knickpoint separately, and it was based on the visual identification of features frequently found on terrestrial knickpoints when a base-level change – e.g. the movement of a tectonic fault or a drop in relative sea level – had formed them. On Earth, a variation in the base-level of a river generates a pulse of vertical incision and channel narrowing, therefore leaving behind abandoned terraces and incised meanders^9,10,11^. Thus, the approach assessed individually their likelihood of being formed by a variation in their base-level is constituted by four aspects: the presence of upslope channel, the identification of vertical incision downstream of the knickpoint, the identification of abandoned terraces downstream of the knickpoint and the identification of incised meanders downstream of the knickpoint. To do that, we used HiRISE (High Resolution Imaging Science Experiment) images with a resolution up to 30 cm per pixel of the region where the knickpoint is located. In areas non-covered by HiRISE, we used the Context Camera (CTX) images with a resolution up to 6 m per pixel. Whilst we acknowledge that there is an on-going debate on whether flood-eroded channels can form inner strath terraces (exemplified in the case of the Channeled Scabland (see Ref 20 & 48)), we are inclined to classify as strath/abandoned terraces those adjacent surfaces downstream a major knickpoint. If only because of the massive dimensions of such knickpoints. We created a table with four questions per knickpoint, we answered each question as “yes” or “no” for each knickpoint individually. Those knickpoints with two or more “yes” as answers were classified as possible base-level fall knickpoints. We used the following questions:

- Do you identify upslope channel?

- Do you identify a new pulse of vertical incision?

- Do you observe abandoned terraces downstream the knickpoint?

- Do you observe incised meanders downstream the knickpoint?

We created a database with all the base-level change knickpoints. The database comprises the ID number of the base-level change knickpoint, the name of the channel system in which was identified, the elevation (extracted from the sinkless raster) of the initial point of the reach in which the bottom surface of the knickpoint was located, the elevation (extracted from the original raster) of the initial point of the reach in which the bottom surface of the knickpoint was located, the coordinates of the initial point of the reach in which the bottom surface of the knickpoint was located and the number of “yes” answers for each knickpoint. In the table 1, we can see the 34 candidates selected as base-level change knickpoints. Those rows with their background colored (blue, brown, green and red) represent the knickpoints located within a knickpoint zone displayed in the figure 2 of the manuscript. We assigned the same color to each knickpoint in the table 1 shown below as in the figure 2 of the manuscript.

Several knickpoints sharing a similar elevation of their bottom surface suggest that they might come from a common perturbation^8,18^. The more widespread they are, the less likely that perturbation is related to local factors (as local lithology or tectonic uplifts). Hence, a global assessment is of significant importance for the aim of the present study. We will explain the global assessment performed in the following section.

TABLE 1. DATABASE WITH THE BASE-LEVEL CHANGE KNICKPOINTS

| ID | Channel system | Bottom elevation^1^ (m) | Bottom elevation^2^ (m) | Coordinates | Yes |
| --- | --- | --- | --- | --- | --- |
| 1 | Abus Valley | -2159 | -2194 | 5°21'58''S 147°12'20''W | 3 |
| 2 | Abus Valley | -2496 | -2510 | 5°00'06''S 147°14'07''W | 2 |
| 3 | Al-Qahira | -20 | -24 | 21°37'36''S 159°43'11''E | 3 |
| 4 | Al-Qahira | -643 | -684 | 19°44'34''S 160°52'18''E | 3 |
| 5 | Al-Qahira | -976 | -977 | 18°54'56''S 161°39'02''E | 2 |
| 6 | Al-Qahira | -1184 | -1200 | 18°16'45''S 161°56'29''E | 3 |
| 7 | Bahram Valles | -959 | -965 | 20°33'25''N 59°06'60''W | 2 |
| 8 | Bahram Valles | -1299 | -1352 | 20°50'24''N 58°08'25''W | 2 |
| 9 | Kasei Valles Northern branch | -2445 | -2502 | 26°02'46''N 70°32'33''W | 3 |
| 10 | Kasei Valles Northern branch | -2827 | -2845 | 26°34'09''N 62°32'39''W | 2 |
| 11 | Kasei Valles Northern branch | -2877 | -2877 | 26°15'47''N 61°44'12''W | 2 |
| 12 | Kasei Valles Northern branch | -3200 | -3200 | 27°22'59''N 60°46'33''W | 3 |
| 13 | Kasei Valles Southern branch | -2010 | -2029 | 18°20'43''N 74°09'12''W | 4 |
| 14 | Kasei Valles Southern branch | -2374 | -2463 | 20°45'50''N 72°39'17''W | 4 |
| 15 | Kasei Valles Southern branch | -2751 | -2763 | 21°14'28''N 72°09'42''W | 3 |
| 16 | Kasei Valles Southern branch | -3470 | -3471 | 25°07'43''N 61°09'19''W | 4 |
| 17 | Licus Valles | 900 | 892 | 03°51'03''S 126°43'51''E | 3 |
| 18 | Licus Valles | 812 | 800 | 02°58'06''S 126°21'54''E | 3 |
| 19 | Licus Valles | -1695 | -1708 | 00°26'47''N 126°51'01''E | 2 |
| 20 | Licus Valles | -2323 | -2324 | 00°35'16''N 131°09'46''E | 3 |
| 21 | Maja Valles | -1061 | -1073 | 16°16'43''N 57°20'37''W | 3 |
| 22 | Maja Valles | -1212 | -1212 | 16°45'30''N 56°34'06''W | 3 |
| 23 | Maja Valles | -2141 | -2162 | 17°09'48''N 55°20'52''W | 3 |
| 24 | Maja Valles | -2438 | -2461 | 17°42'10''N 54°12'53''W | 3 |
| 25 | Maja Valles | -3412 | -3441 | 20°15'37''N 49°59'26''W | 3 |
| 26 | Mangala Valles | -1287 | -1315 | 07°35'20''S 151°07'11''W | 2 |
| 27 | Mangala Valles | -2569 | -2626 | 05°17'47''S 150°27'15''W | 3 |
| 28 | Mawrth Valles | -3354 | -3384 | 23°38'16''N 18°28'51''W | 3 |
| 29 | Mawrth Valles | -3460 | -3460 | 24°28'40''N 18°31'47''W | 3 |
| 30 | Minio Valles | -650 | -650 | 09°17'41''S 151°10'11''W | 2 |
| 31 | Minio Valles | -2092 | -2108 | 05°00'39''S 151°57'42''W | 2 |
| 32 | Minio Valles | -2604 | -2620 | 04°19'34''S 151°38'22''W | 3 |
| 33 | Shalbatana Valles | -3019 | -3040 | 04°45'35''N 44°01'41''W | 3 |
| 34 | Ares Valles | -3470 | -3470 | 02°08'02''N 17°30'15''W | 3 |

^1^Elevations of the bottom surface of the knickpoint extracted from the sinkless DEM

^2^Elevation of the bottom surface of the knickpoint extracted from the original DEM

**4. Comparison of knickpoint elevation and distribution across the planet**

As knickpoints migrate upslope, their elevation rises gradually from their originating base-level (see figure 1 in the manuscript)^15^. Thus, knickpoints from a same base-level change on separate valleys may have slightly different elevations^15^ and, therefore, to look for commonality in the elevation of such base-level change knickpoints, we need to look over a range of elevations. Such an interval can be defined as range of elevations within which Martian knickpoints coming from the same perturbation lie.. The first step in the global assessment was the definition of 9 Gaussian Kernel Functions, each of them developed with a different bandwidth value. We selected as bandwidth values those corresponding to 30m, 60m, 90m, 120m, 150m, 210m, 270m, 330m and 420m in elevation.

For each of the bandwidth values, the elevation at the bottom surface of each knickpoint from the original DEM was used. We added and subtracted width/2 (15m in the case of 30m width value) to this elevation, therefore obtaining an interval for each candidate: its elevation ±width/2. Subsequently, we estimated the Kernel Function of all the elevations within its interval by a normal distribution function with amplitude 1 in the center of the interval and 0 in its boundaries. To generate that, we used the function “Norm.Dist()” in excel, defining the value corresponding to each elevation by calculating the distance between that elevation and the center of the interval, and assigning to that value the corresponding normal distribution with a mean equal to 0.05 and the standard variable (SD) worked out by equation 2:

SD = 3 + bandwidth/10 (2)

Following this, the Kernel Density Estimate was determined by summing, per elevation, the value of each Kernel function. We repeated the same process with each bandwidth value, eventually obtaining 9 Kernel Density Estimates with different smoothness degrees as seen below in the figure 3. As each base level change knickpoint is therefore represented by a Gaussian Kernel Function of less or equal than one, if there is a summed Kernel Density Estimate greater than one it means there is more than one base-level change knickpoint concurrent around that elevation.

We defined as knickpoint zones those elevations in which the summed Kernel Density Estimate exhibits a frequency value that is not inherent in the record. Put simply, a value that has not been obtained in every bootstrap resample performed in the section below. For a Gaussian width of 90 m, a knickpoint zone is defined by a minimum frequency value of 1.4 (Supp. Fig. 12). In addition, at least one of the knickpoint comprising this commonality has to be in a different longitudinal zone from the others. E.g. three knickpoints found at the same elevation: two of them located in two neighbor channels and the other in a distant channel. In this case, the cluster of knickpoints constitutes a knickpoint zone, since the likelihood of a local factor affecting a wide range of longitudes is low. Thus, the similar elevation and widespread locations of knickpoints within a knickpoint zone suggest common global controls.

**Assessing the probability of local lithologic controls**

The alternative forming mechanism is by high fluvial discharges over layers of different resistance bedrock^18^. Reasonably, unless the formation of the Martian crust resulted in planet wide layers of resistant material, these lithologic controls can be assumed to be local and, therefore, to vary randomly across the planet. On this basis, the odds of these knickpoints occurring at similar elevations can be assessed as the probability that random changes in the local lithology of the 12 rivers generated 34 knickpoints mimicking the altitudinal distribution of our record. Hence, we applied a Bootstrapping method to assess the likelihood of this alternative hypothesis.

First, we used our original sample of 34 knickpoints within these 12 long profiles to generate repeated (1,000) resamples with replacements. For this, we associated a random number – different for each iteration – between 1 and 4 with each long profile, thus defining the number of knickpoints within each long profile and summing 34. Based on these random numbers, we generated the corresponding number of knickpoints per stream by randomly selecting elevations within the altitude of each long profile (e.g. if Maja Valles obtained 3 knickpoints and extends from -500 to -3900m, we generated 3 random numbers between -500 and -3,900). We repeated this procedure 1,000 times, thereby bootstraping our original sample with different resamples. Subsequently, we calculated the Kernel Density Estimate for each resample by following the same procedure explained above (section 4). We used 3 different bandwidths (corresponding to 30m, 90m and 150m), thus obtaining 3 different Kernel Density Estimates per resample. Finally, we calculated the mean commonality of knickpoint for each resample and bandwidth by extracting all the peaks within the Density Function and averaging their values. Thus, enabling us to produce a bootstrap distribution of resampled means for each bandwidth. We worked out the mean and 95% bootstrapping confidence interval for each distribution to assess the significance of non-random processes in the nature of our record of knickpoints (Supp. Fig. 9).

Importantly, to assess the significance of non-random processes in the generation of our zones 3 & 4, we split our record into 2 sub-records (one sub-record composed by the 8 knickpoints and 5 long profiles comprising the zones 3 & 4 and the other composed by the rest of knickpoints and long profiles). We applied the same procedure as above to each sub-record, thereby producing a boostrap distribution of resampled means for each sub-record. Finally, we worked out the mean and 95% bootstrapping confidence interval to assess the significance of non-random processes in the nature of each sub-record (Supp. Fig. 10 & 11).

**Generation of the Supplementary Figure 7**

In this figure, we imported into Photoshop the original image showing the Arabia, Deuteronilus and Isidis Shorelines, together with the contribution of Tharsis to Mars’ topography from (Citron, R.I., et al., 2018). On top of the image, we added the base-level change knickpoints within knickpoint zones proposed in this paper. We represented each knickpoint as a point with the same colour as the knickpoint zone in which is located (see figure 2 in the manuscript). As can be seen in the figure 7 displayed below, knickpoints from the knickpoint zone 1 and 2 (brown and green points) are located in regions where the contribution of Tharsis to Mars’ topography is estimated to be low. Thus, they could have been formed by a variation of the level of an ocean before (or during the early stages of) Tharsis emplacement and still preserve their initial elevation. On the contrary, knickpoints from the knickpoint zone 3 (blue) are located in regions where different levels of contribution of Tharsis to Mars’ topography are estimated. Hence, it suggests they possibly formed by a variation of the level of an ocean after (or during the latter stages of) Tharsis emplacement.

**REFERENCES CITED**

48. Baker, V.R., 1978. The Spokane Flood controversy and the Martian outflow channels: *Science*, v. 202, p. 1249 1256.


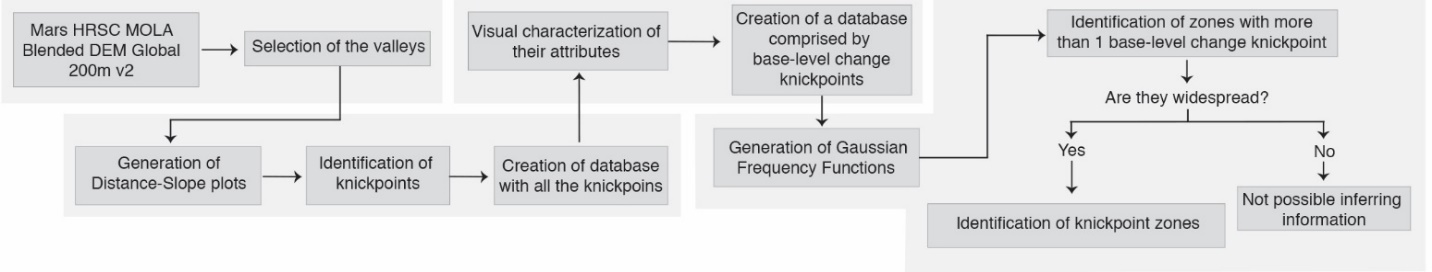


**Suppl. Fig. 1. - Methodological approach followed in the present paper.** The methodological approach contains 4 phases: selection of the channels, identification of knickpoints, identification of base level change knickpoints and comparison of knickpoint elevation and distribution across the planet. Each phase is contained within a grey square. The arrows indicate the flow of our approach.


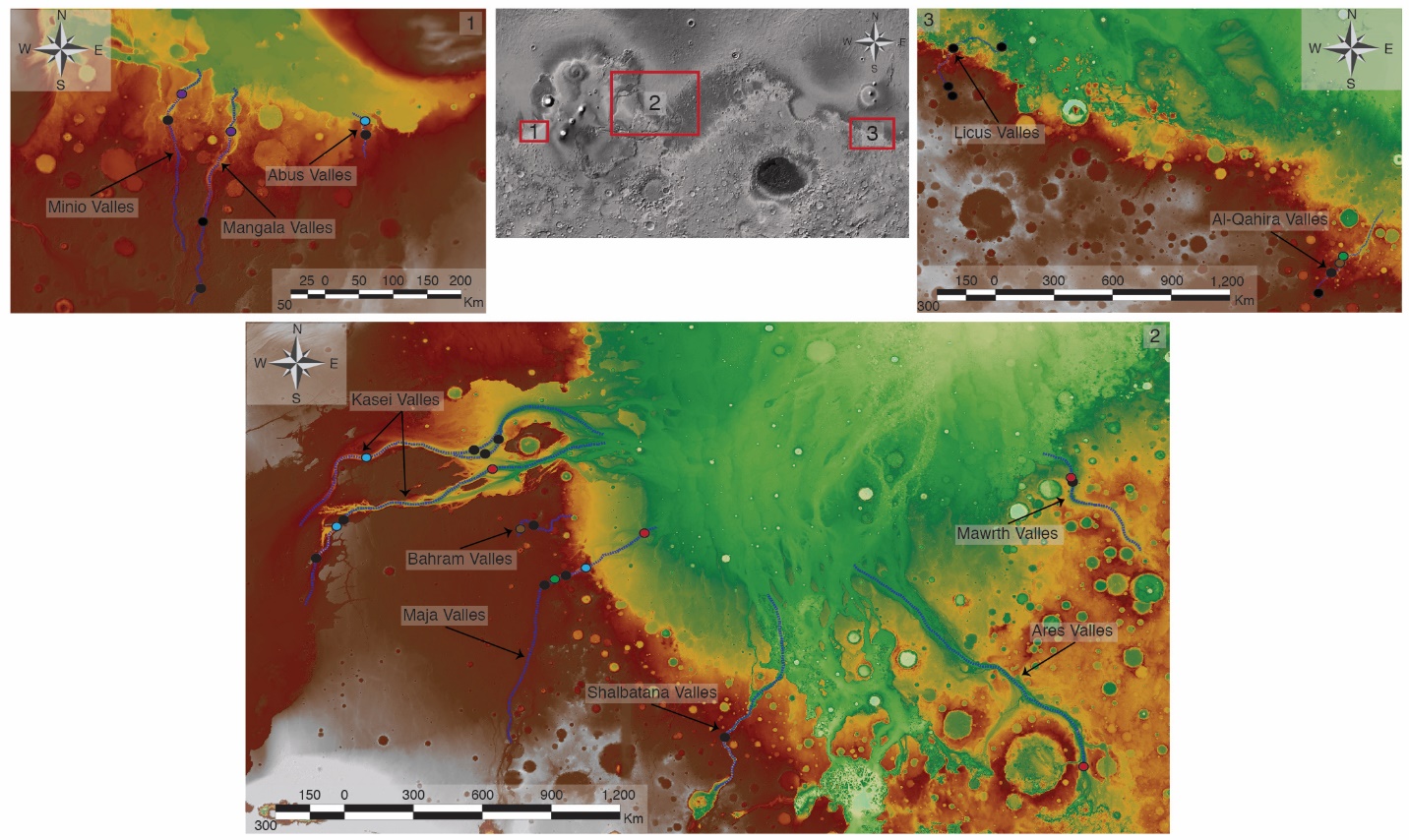


**Suppl. Fig. 2. – Location of the 34 base-level change knickpoints zones in the Martian channels**. Each knickpoint is represented by a point with a different color, depending on the knickpoint zone is located (see figure 2 in the manuscript). The figure also contains the name of the channel systems, a scale and a global map displaying the location of each sub-figure. The images for these panels (1, 2 & 3) are color-coded shaded-relief MOLA digital elevation models (460m/pixel). Credit: MOLA Science Team, MSS, JPL, NASA. We produced the mosaic and maps in this figure using Esri’s ArcGIS 10.6 software (http://www.esri.com/software/arcgis).


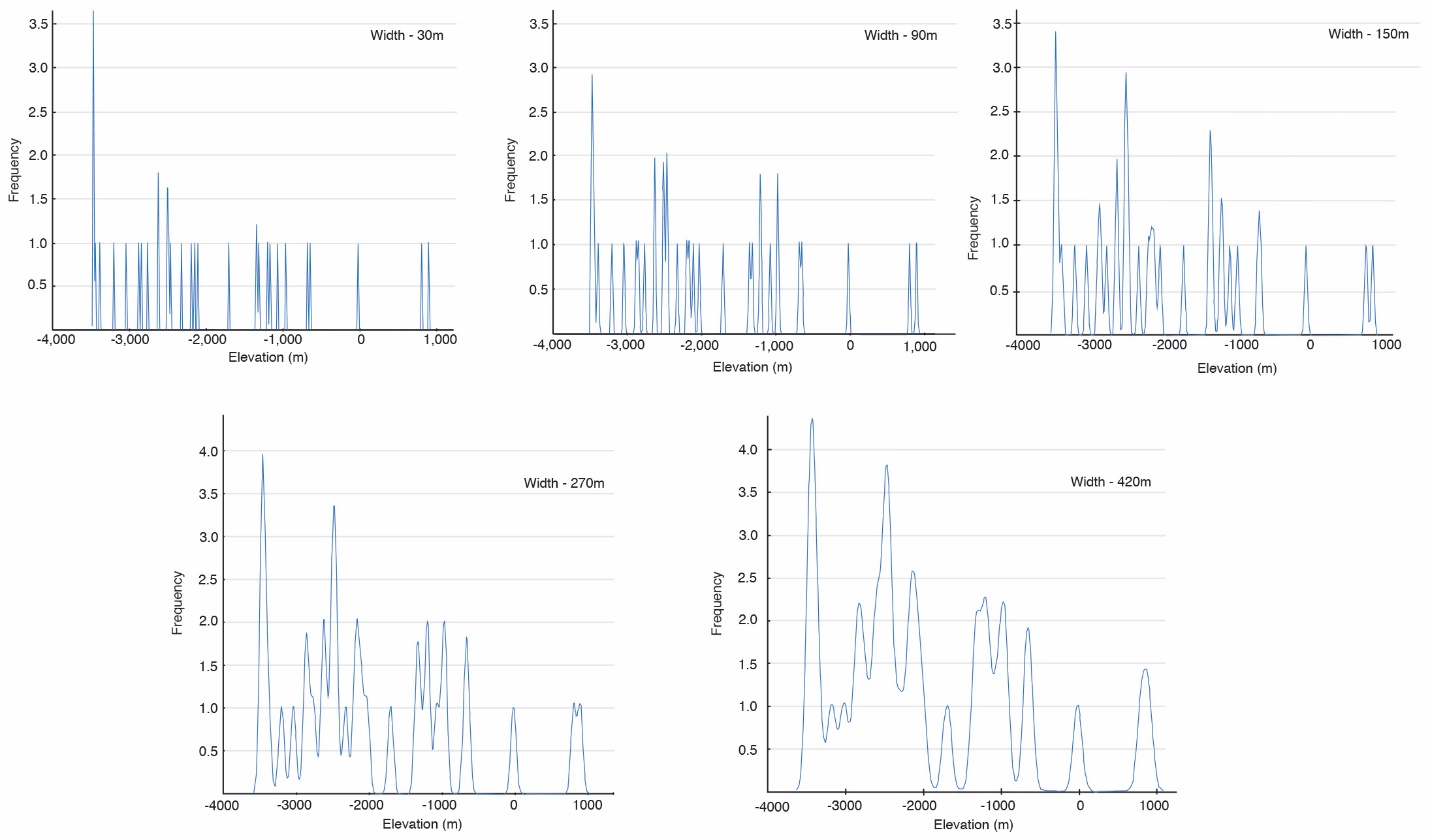


**Suppl. Fig. 3. – Kernel Density Estimates of the concentration per elevation of the 34 base-level change knickpoints, obtained by 5 different bandwidth values**. Density functions displaying the concentration of base-level change knickpoints per elevation obtained for bandwidth values corresponding to 30m, 90m, 150m, 270m and 420m in elevation. We selected the bandwidth value corresponding to 90 m in elevation since it optimally smoothes the Kernel Density Estimate. Kernel functions with 60m, 120m and 210m bandwidth values were also included in the assessment, although they are not present in this figure.


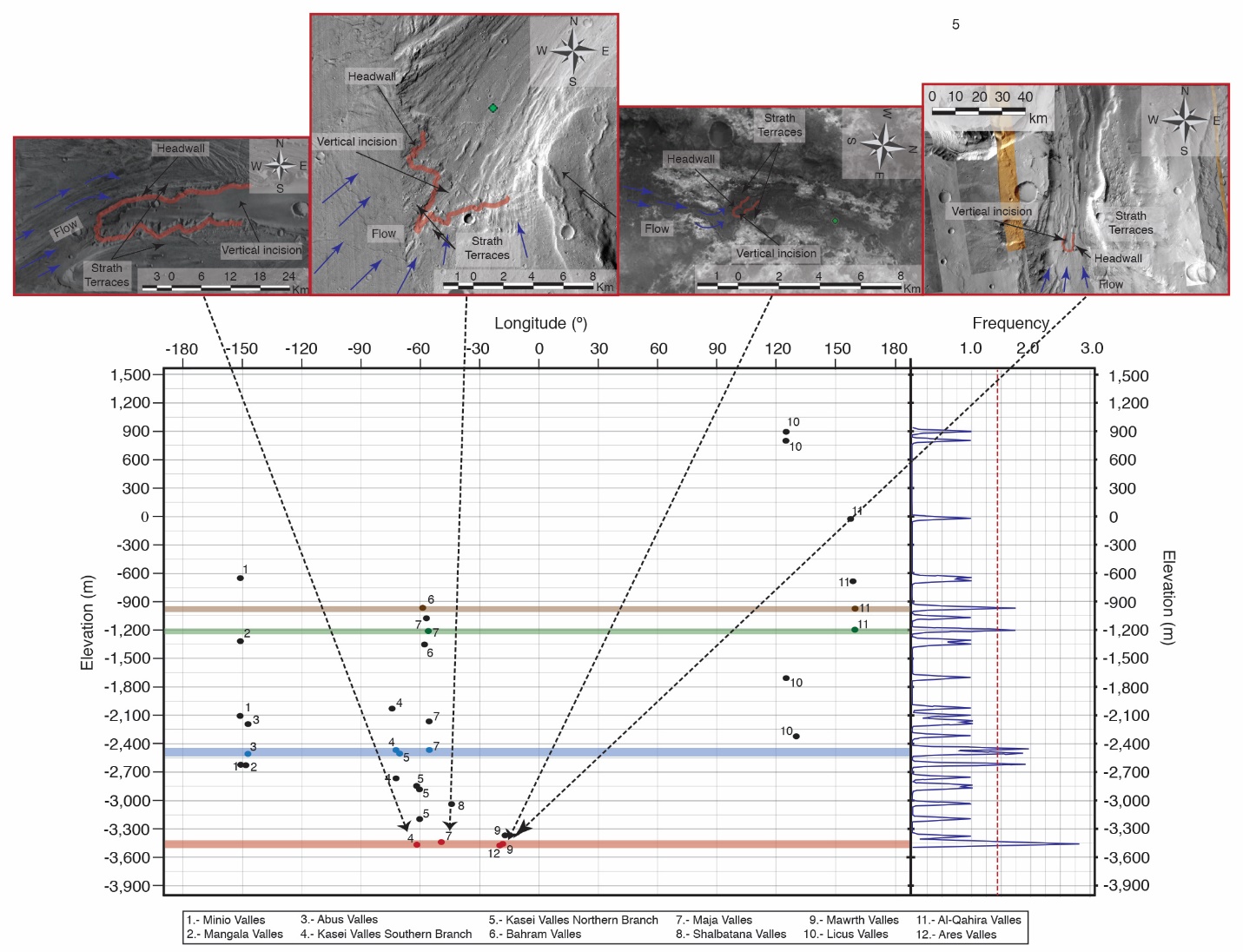


**Suppl. Fig. 4. – Aerial sketched images of the knickpoints composing the knickpoint zone 4**. Aerial HiRISE and CTX images of the base-level change knickpoints found in the knickpoint zone 4. Each image has been sketched with the detected features that typically a base-level change knickpoint exhibits. From left to right, they are the candidate knickpoints number 16, 25, 29 in the table 1.

**
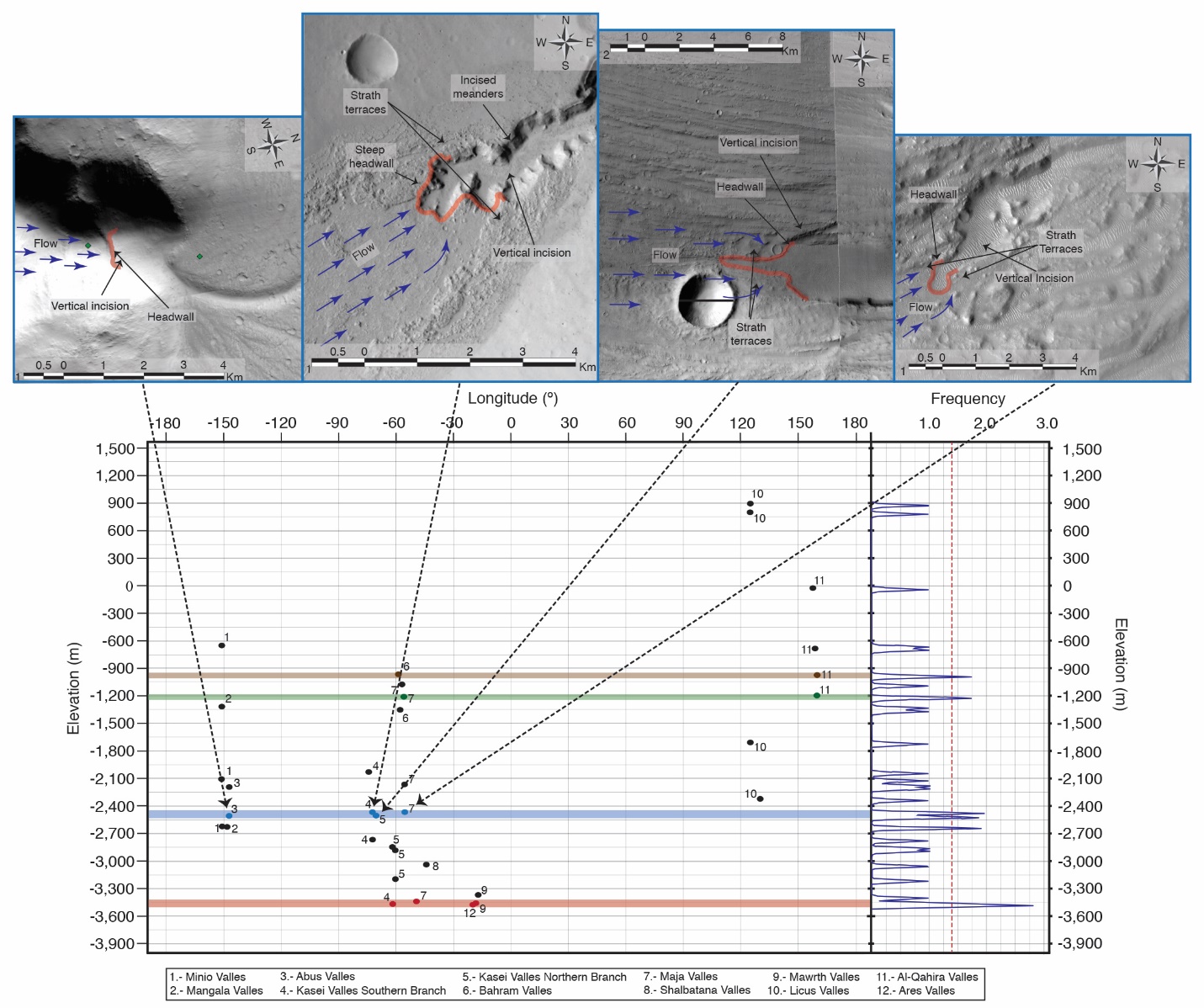
**

**Suppl. Fig. 5. – Aerial sketched images of the knickpoints composing the knickpoint zone 3.** Aerial HiRISE and CTX images of the base-level change knickpoints found in this knickpoint zones. Each image has been sketched with the detected features that typically a base-level change knickpoint exhibits. From left to right, the images displays the candidate knickpoints numbered as 2, 9, 14 in the table 1.


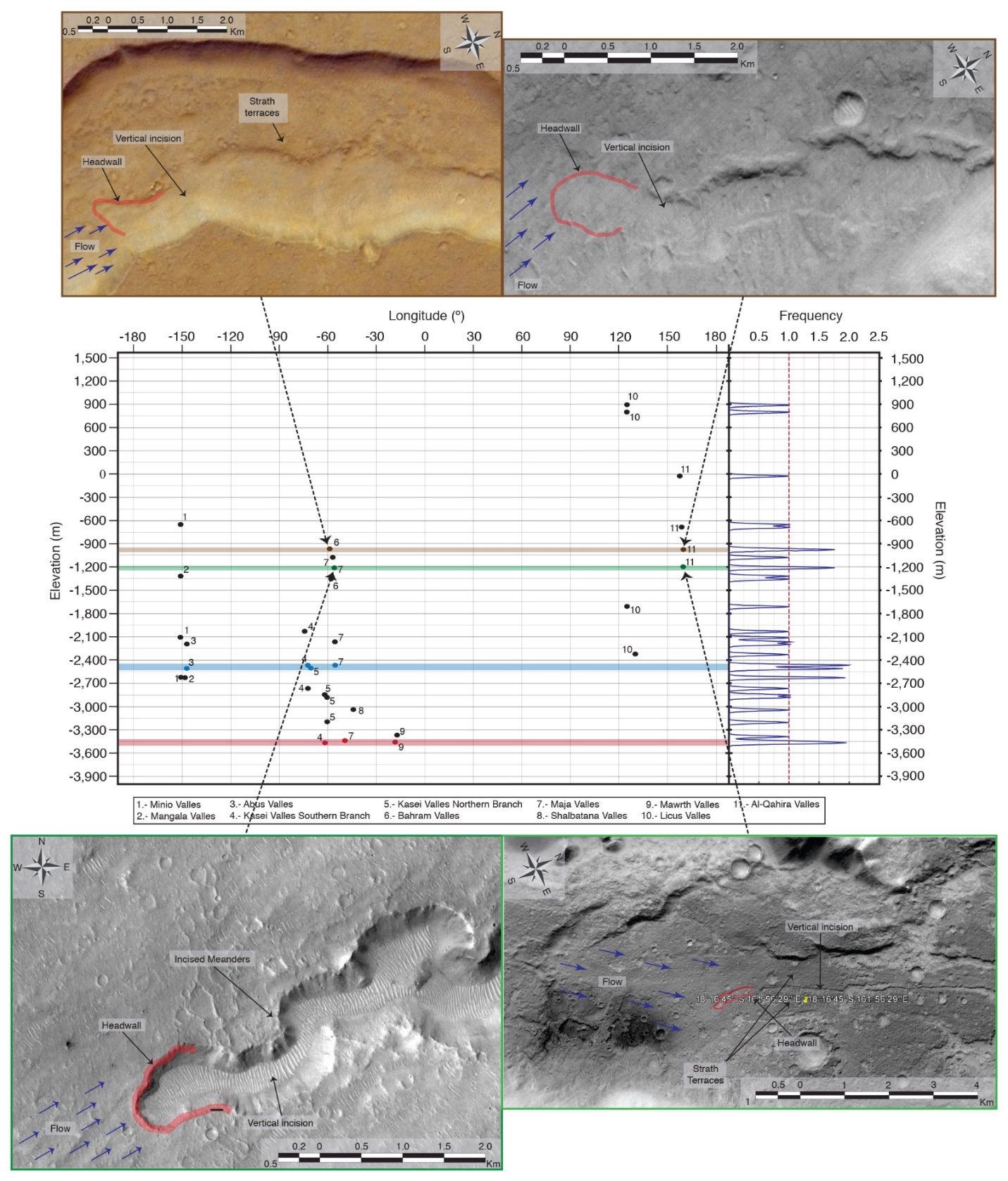


**Suppl. Fig. 6. – Aerial sketched images of the knickpoints composing the knickpoint zone 1 and 2.** Aerial HiRISE and CTX images of the base-level change knickpoints found in such knickpoint zones. Each image has been sketched with the detected features that typically a base-level change knickpoint exhibits. From left to right, the images on top display the candidate knickpoints numbered as 5 and 7 in the table 1. Likewise, the images at bottom display the candidate knickpoints numbered as 6 and 22 in the table 1.


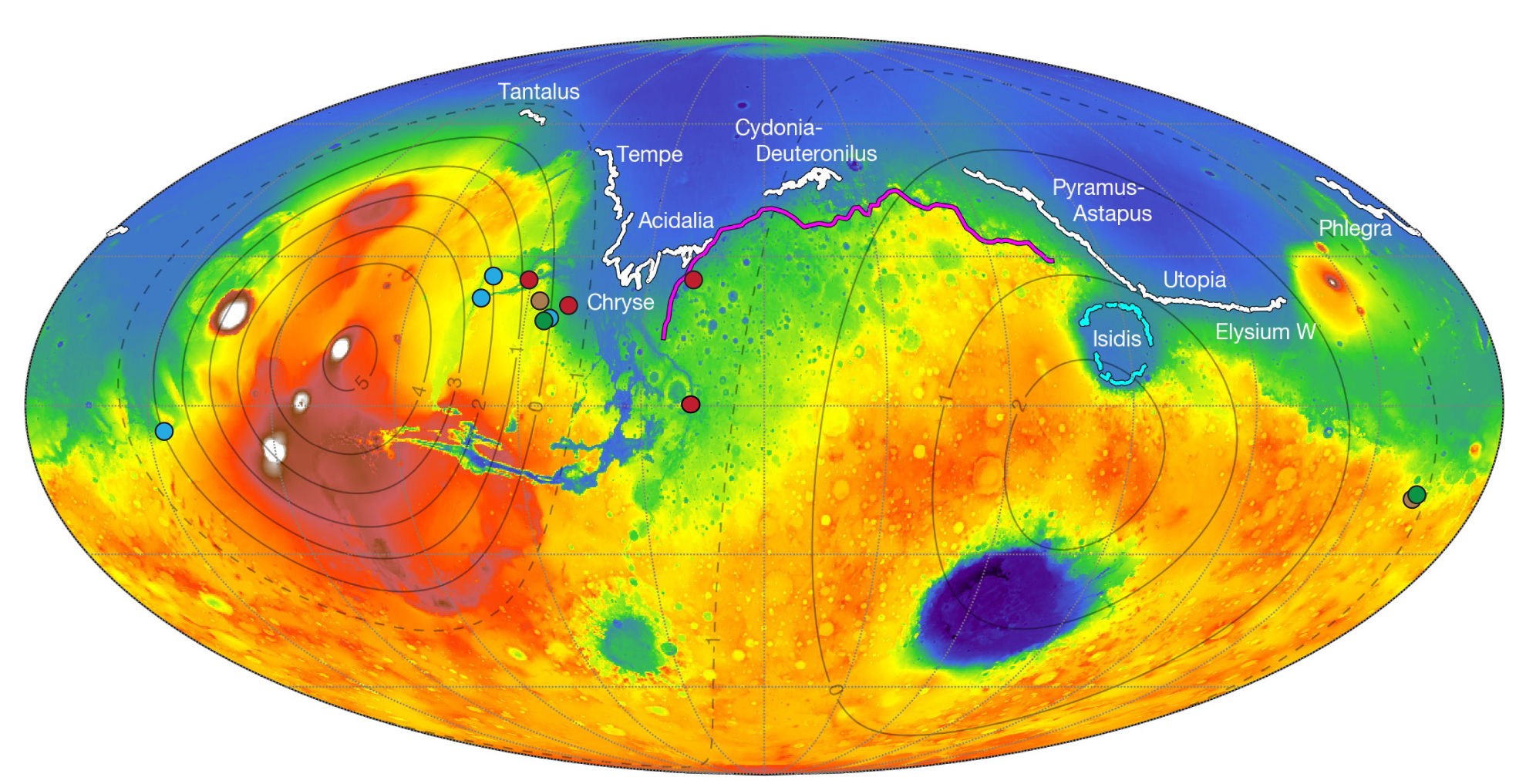


**Suppl. Fig. 7. – Map of the shoreline location, MOLA topography, Tharsis deformation and location of the base-level change knickpoints within knickpoint zones**. The Arabia, Deuteronilus and Isidis Shorelines, together with the contribution of Tharsis to Mars’ topography are from the reference 29. Such contribution of Tharsis is ranked up to degree-5, the negative contribution is displayed as a dashed line. The base-level change knickpoints located within knickpoint zones are represented by points. The color of the point indicate the knickpoint zone in which is located (see figure 2 in the manuscript). The basemap was reprinted by permission from “Springer Nature: Timing of Oceans on Mars from Shoreline Deformation, v. 555, p. 643-646, Citron, R., Manga, M. & Hemingway, D.J. Copyright 2018.”


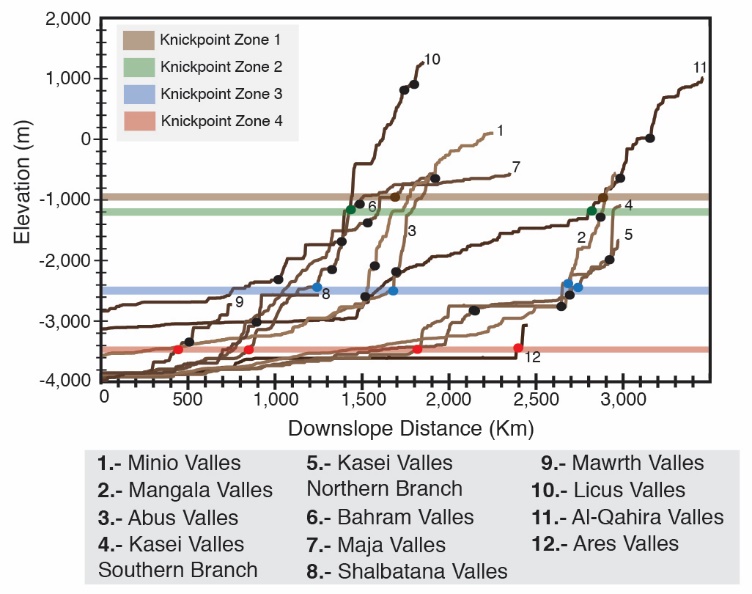


**Suppl. Fig. 8. - Longitudinal profiles of the Martian channels, location of potential base-level change knickpoints and knickpoint zones**. The graph shows the longitudinal profiles of the Martian channels associated with a number that identifies the channel. The graph incorporates the location of the possible base-level change knickpoints. Each base-level change knickpoint is represented by a point with the same colour than the knickpoint zone in which is included. Black knickpoints are not contained within any knickpoint zone. The longitudinal profiles were extracted from a sinkless raster.


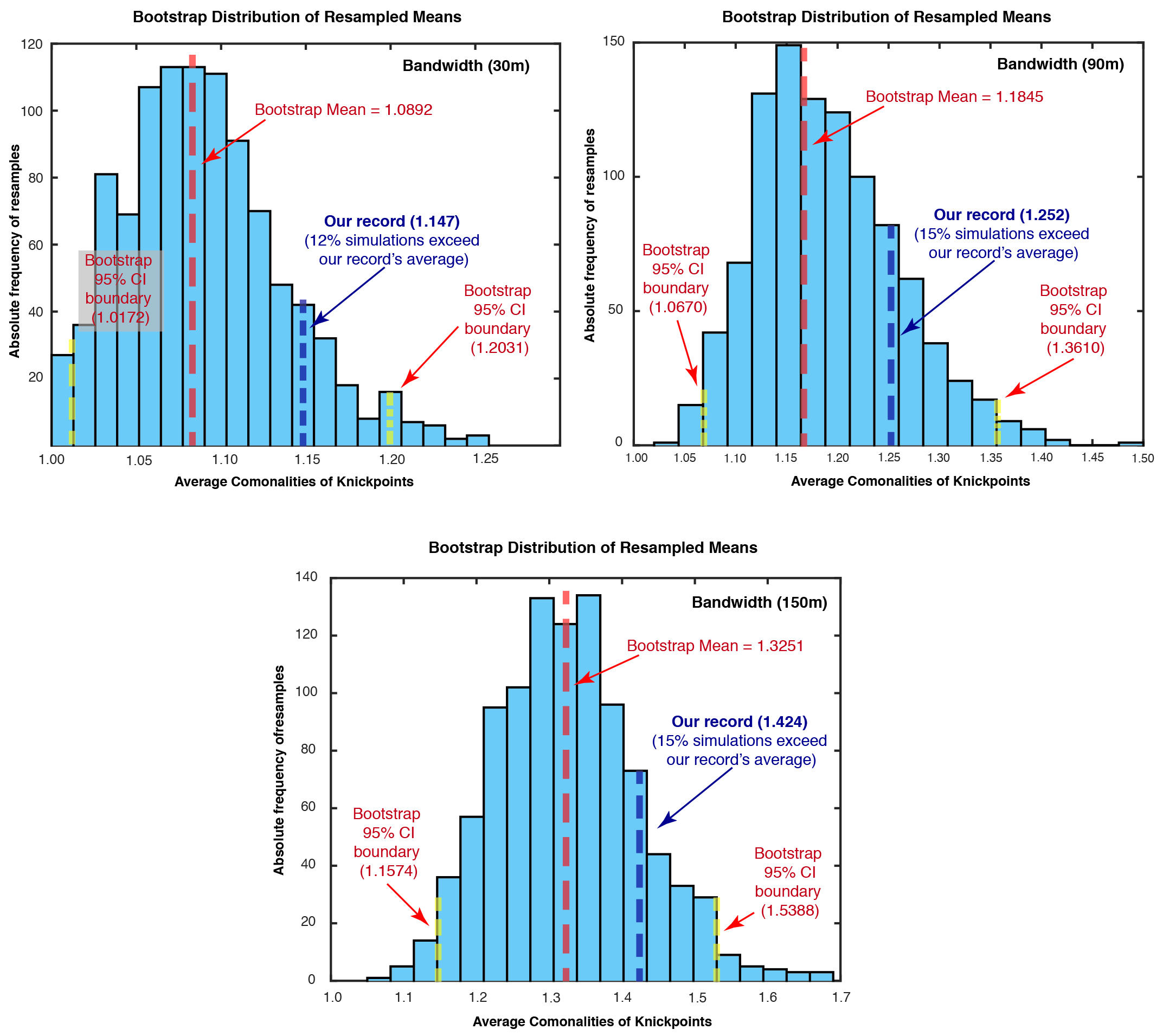


**Suppl. Fig. 9. – Bootstrap Distribution of Resampled Means for our record of 34 knickpoints**. The graph shows the distribution of resampled means obtained by bootstrapping our sample of 34 knickpoints. For each of the three distributions, we have used a different bandwidth to work out the Kernel Density Estimate. Each distribution displays the bootstrap mean and 95% confidence interval, as well as the average commonality of knickpoints with our record.


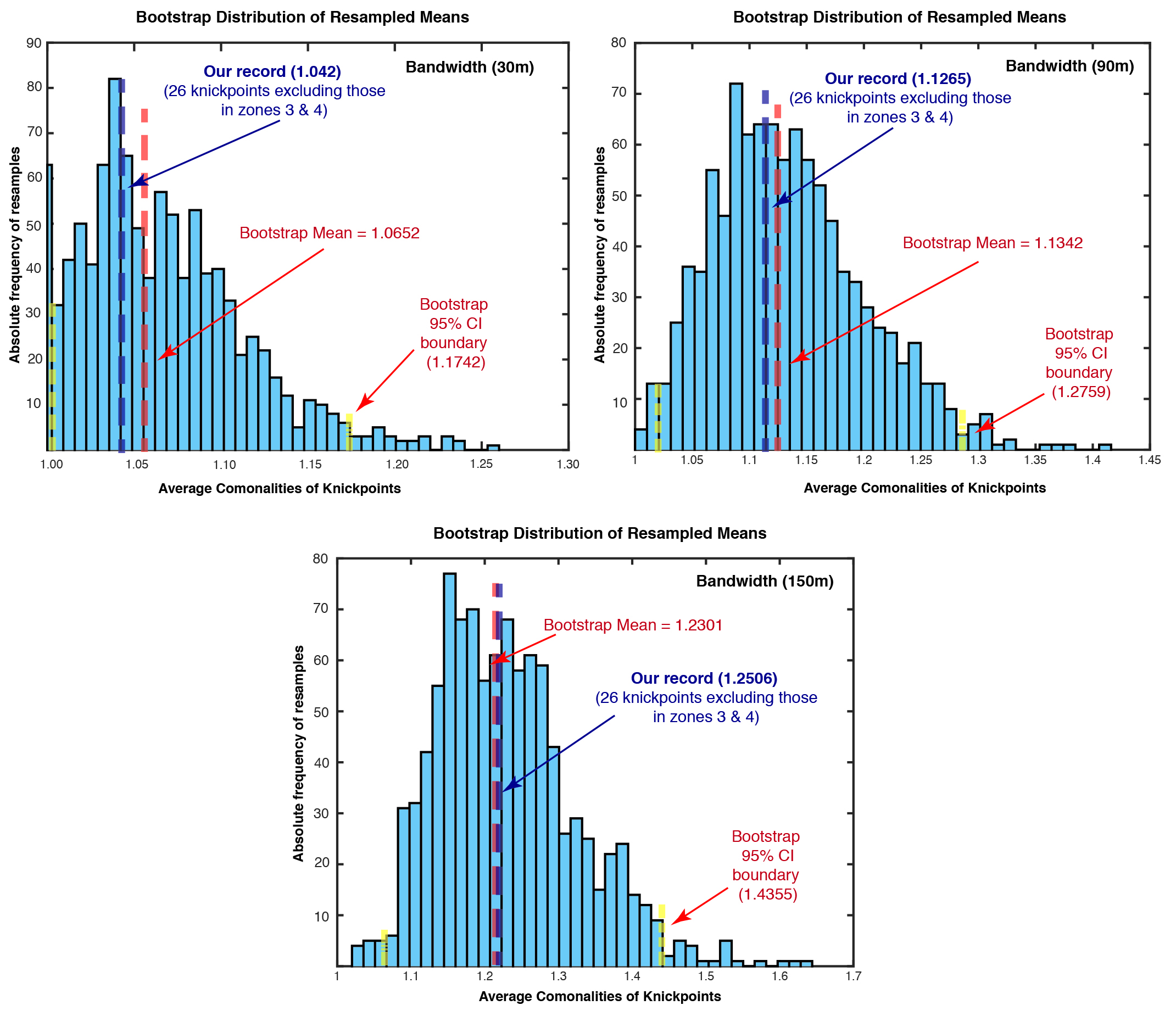


**Suppl. Fig. 10. – Bootstrap Distribution of Resampled Means for our sub-record of 26 knickpoints in zones 1 and 2**. The graph shows the distribution of resampled means obtained by bootstrapping our sample of 26 knickpoints. For each of the three distributions, we have used a different bandwidth to work out the Kernel Density Estimate. Each distribution displays the bootstrap mean and 95% confidence interval, as well as the average commonality of knickpoints with our sub-record.


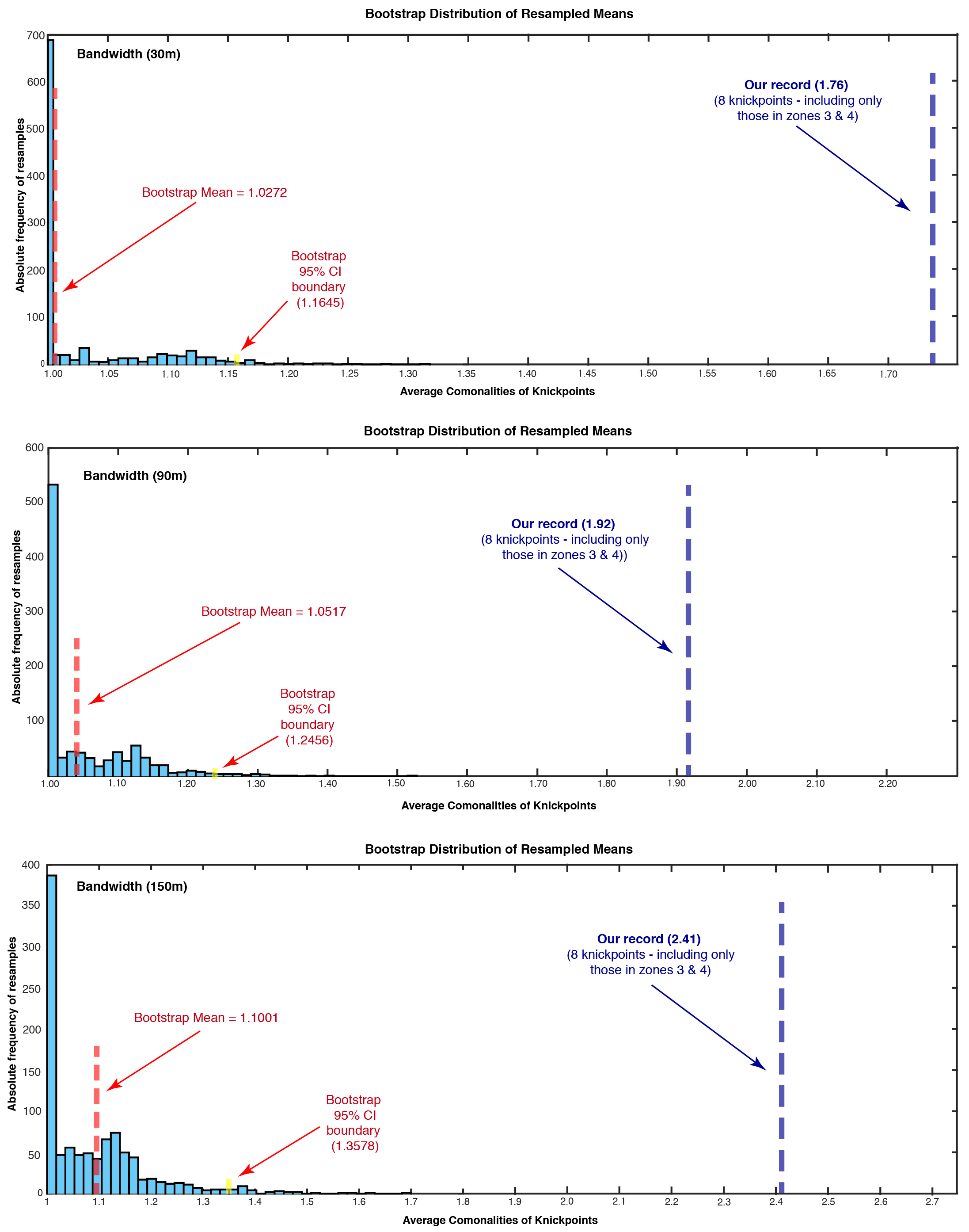


**Suppl. Fig. 11. – Bootstrap Distribution of Resampled Means for our sub-record of 8 knickpoints within zones 3 & 4**. The graph shows the distribution of resampled means obtained by bootstrapping our sample of 8 knickpoints. For each of the three distributions, we have used a different bandwidth to work out the Kernel Density Estimate. Each distribution displays the bootstrap mean and 95% confidence interval, as well as the average commonality of knickpoints with our sub-record.
